# Does Automated Versus Manual Ki67 Labeling Index Assessment Influence Risk Stratification in Patients with Localized Adrenocortical Carcinoma? Lessons from the ADIUVO Trial

**DOI:** 10.3390/cancers18162678

**Published:** 2026-08-18

**Authors:** Marijn A. Vermeulen, Linde M. Amelung, Otilia Kimpel, Ulrich Dischinger, Vittoria Basile, Darko Kastelan, Hélène Lasolle, Isabelle Bourdeau, Svenja Nölting, Paola Loli, Magalie Haissaguerre, Alfredo Berruti, Martin Fassnacht, Massimo Terzolo, Ronald R. de Krijger

**Affiliations:** 1 Princess Máxima Center for Pediatric Oncology, 3584CS Utrecht, The Netherlands; 2 University Medical Center Utrecht, 3584CX Utrecht, The Netherlands; 3 Department of Internal Medicine, Division of Endocrinology and Diabetes, University Hospital, University of Wurzburg, 97080 Wurzburg, Germany; 4 Internal Medicine, Department of Clinical and Biological Sciences, San Luigi Gonzaga Hospital, University of Turin, 10043 Orbassano, Italy; 5 Department of Endocrinology, University Hospital Zagreb, 10000 Zagreb, Croatia; 6 Federation d’Endocrinologie, Hospices Civils de Lyon, University de Lyon, 69500 Bron, France; 7 Service d’Endocrinologie, Département de Médecine, Centre Hospitalier de l’Universite de Montréal (CHUM), Montreal, QC H2X 3E4, Canada; 8 Department of Endocrinology, Medizinische Klinik und Poliklinik IV, Klinikum der Universität München, 80336 Munich, Germany; 9 Endocrinology, Clinica San Carlo, 20037 Milano, Italy; 10 Department of Endocrinology and Endocrine Oncology, Haut Leveque Hospital, University Hospital of Bordeaux, 33604 Bordeaux, France; 11 Medical Oncology, ASST-Spedali Civili, University of Brescia Comprehensive Cancer Center, 25123 Brescia, Italy

**Keywords:** adrenocortical carcinoma, Ki67, prognosis, recurrence, survival

## Abstract

Background/Objectives: Adrenocortical carcinoma (ACC) is a rare tumor. Diagnosis relies on combined histopathological criteria that, in a multifactorial scoring system, may suggest malignancy. The recent ADIUVO trial, a multicenter trial randomizing ACC patients between mitotane treatment or surveillance only, demonstrated that patients with localized, low-grade ACC (Ki67 labeling index (LI) ≤ 10%) have a better prognosis than historically anticipated, questioning the routine use of adjuvant mitotane. This post hoc analysis aimed to investigate whether automated, centralized Ki67 LI assessment improves prognostic stratification beyond expert manual assessment in low-risk ACC patients enrolled in the original ADIUVO trial. Methods: Ki67 LI was centrally reassessed in 70 ADIUVO patients by digitizing slides and applying an automated algorithm to manually selected hotspots. Primary endpoints were correlation between methods and the impact of automated scoring on recurrence-free survival (RFS) and overall survival (OS). Results: Automated Ki67 LI assessment was feasible in 47 patients. Manual and automated Ki67 LI values showed good agreement (mean 5.6 ± 3.1% vs. 5.4 ± 5.3%). Automated scoring identified eight patients with KI67 LI > 10%. In this subgroup, RFS and OS did not differ significantly between patients treated with mitotane and those under surveillance. Patients with an automated Ki67 LI > 10% had larger tumors (median 16.0 cm vs. 7.0 cm, *p* = 0.003). Conclusions: Our findings support the original ADIUVO results and support continued use of manual expert Ki67 LI scoring. This traditional approach remains robust and pragmatic for most clinical settings, especially where automated systems are not readily accessible.

## Simple Summary

Adrenocortical carcinoma (ACC) is a rare cancer with a variable risk of recurrence after surgery. The ADIUVO trial showed that patients with localized, low-grade ACC have a favorable prognosis after surgery and do not routinely benefit from adjuvant mitotane treatment. Identifying these low-risk patients relies largely on the Ki67 labeling index (Ki67 LI), making accurate assessment essential. In this post hoc analysis of the ADIUVO trial, we compared expert manual Ki67 LI assessment with a centralized automated digital scoring method. The two approaches showed good agreement, and automated scoring did not improve prediction of patient outcomes beyond expert manual evaluation. Our findings reinforce the original ADIUVO trial results and support continued use of expert manual Ki67 LI assessment, which remains a reliable, practical, and widely available method for risk stratification in patients with low-risk ACC.

## 1. Introduction

Adrenocortical carcinoma (ACC) is a malignancy arising from the cortex of the adrenal gland. With an annual incidence that ranges between 0.7 and 2.0 cases per million individuals, ACC is an uncommon disease that is associated with a generally poor prognosis [1,2,3,4,5]. Diagnosis relies on a combination of histopathological criteria which, in a multifactorial scoring system, indicate malignancy. The most widely used of these scoring systems is the Weiss score, which is applied to ACC in adults with conventional histomorphology [6,7]. For oncocytic variants and ACC in children, other scoring systems have been developed [8,9]. Surgery remains the primary therapeutic approach, with complete resection representing the only curative option [6]. However, even after complete resection, recurrence rates remain high, up to 70%, highlighting the need for adjuvant therapies to reduce relapses and improve long-term outcomes. Current adjuvant therapy options include mitotane, radiotherapy and cytotoxic drugs [6]. Treatment with adjuvant mitotane remains a controversial issue, and mitotane use is compounded by its challenging management and associated toxicity [10,11,12,13]. Furthermore, there is a lack of predictive biomarkers for mitotane response; identifying such markers could spare non-responders from unnecessary treatment-related toxicities [5]. Although radiotherapy diminishes local recurrence risk, it has no definable impact on overall survival [14,15,16]. In all published studies, the analysis of the survival curves of patients with ACC who underwent a radical resection suggests a remarkable heterogeneity in prognosis, with patients who recur very early and others who recur late, underscoring the need for personalized adjuvant therapy. Ki67, an immunohistochemical marker of cellular proliferation, has emerged as the single most important prognostic indicator after an R0 resection of ACC, with higher Ki67 labeling index (LI) correlating with more aggressive disease behavior and poorer outcomes [1,17].

The recent ADIUVO trial evaluated the efficacy and safety of adjuvant mitotane compared to surveillance only in ACC patients stratified at low to intermediate risk of recurrence [18]. Risk stratification was based on a widely accepted prognostic scheme incorporating ACC stage, resection status and Ki67 LI. Patients were included in the trial if they had localized disease (stage I to III ACC), microscopic margin-free resection (R0), and low-grade tumor (Ki67 LI ≤ 10%). ADIUVO showed that the survival of such patients was much better than anticipated, with a 5-year recurrence-free survival (RFS) approximating 75% [18]. This trial did not show a significant difference in RFS between patients who received mitotane or active surveillance only, arguing against the routine use of mitotane as an adjuvant measure in patients with low-grade, localized ACC that has been completely resected.

In this post hoc analysis of the ADIUVO trial, we evaluated the impact of standardized, centralized automated Ki67 LI scoring in low-risk ACC patients. By comparing automated measurements to conventional expert manual assessments, we aimed to address inter-center variability and determine whether automated Ki67 LI scoring improves risk stratification and the prediction of patient outcomes [19].

## 2. Materials and Methods

### 2.1. Study Design and Participants

We performed a centralized re-assessment of Ki67 LI of patients enrolled in the ADIUVO study, which included an open-label, phase-3, randomized trial and a parallel, prospective, non-randomized study. Both studies included individuals with low to intermediate-risk ACC (resection status R0, ENS@T stage I-III, Ki67 LI ≤ 10%, based on localized reading) who were managed at the same centers in an identical manner. Treatment allocation was randomized in the interventional trial, while it followed patient’s choice in the observational study that included patients who refused randomization in the trial. Detailed description of the treatment approaches of this trial is given in the original paper [18]. Data from the original pathology reports, including manually assessed Ki67 LI, were collected.

Formalin-fixed paraffin-embedded tissue specimens including blocks, unstained slides or the original Ki67-stained slides were sent to University Medical Center Utrecht, Utrecht, The Netherlands, for re-evaluation of Ki67 LI.

### 2.2. Ethical Approval

The study adhered to the principles of the Declaration of Helsinki and the Good Clinical Practice Guidelines and was approved by the ethics committee at each study center (approval at the coordinating center on 21 January 2008; reference number 153/INT). All patients provided written informed consent to participate.

### 2.3. Ki67 Assessment

Ki67 immunohistochemistry was performed on a BOND-III automated stainer (Leica Biosystems, Wetzlar, Germany) using the ready-to-use Ki67 antibody from Leica (clone MM1). Slides were then digitized in an Aperio slide scanner (Aperio GT450 DX scanner, Leica Biosystems Imaging, Nussloch, Germany) and stored in the Picture Archiving and Communication System (PACS, Sectra, Linkoping, Sweden) at the Princess Máxima Center for Pediatric Oncology, Utrecht, The Netherlands. Eleven cases had Ki67 immunohistochemistry performed elsewhere. These Ki67 slides were received and scanned at the Princess Máxima Center for Pediatric Oncology. To determine the Ki67 LI, an expert endocrine pathologist (RK) manually selected hotspot regions on the digitized slides, which were subsequently analyzed using the standard custom algorithm provided by Sectra. The pathologist (RK) performing the Ki67 assessment was blinded to the Ki67 LI score obtained in the ADIUVO trial.

### 2.4. Outcomes

The ADIUVO trial included 91 patients in the randomized trial and 95 patients in the observational study. Data and materials were obtained from 53 patients in the randomized trial and 17 patients in the observational study, totaling 70 patients.

The primary endpoint was to compare automated Ki67 LI with individual center-based scoring methods. Patients with Ki67 LI scores exceeding 10% via automated scoring were compared to those documented in the ADIUVO trial, where the inclusion criteria required a Ki67 LI ≤ 10%. Subsequent analyses evaluated RFS and overall survival (OS) in these patients, particularly within the subgroup receiving mitotane. RFS was defined as the time from randomization (mitotane versus surveillance) in the randomized trial, or from the time of registration in the observational study, to the first evidence of recurrence. OS was defined as date of randomization, or registration in the study, to date of death due to any cause. Patients without recurrence or death were censored at the date of the last follow-up point.

### 2.5. Statistical Analysis

Statistical calculations were performed using SPSS for Windows version 30. *p*-values (two-sided) < 0.05 were regarded as significant. For correlation between manual and automated Ki67 LI, the paired *t*-test was used. For comparison of Ki67 LI (cut-off 10%) and recurrences, death and stage, the Fisher’s exact test was used and for comparison with tumor diameter, the Mann–Whitney U test was performed. Survival analyses were carried out by plotting a Kaplan–Meier survival curve and assessing significance with log-rank test.

## 3. Results

We obtained material and the corresponding original pathology reports from 70 patients enrolled in the ADIUVO trial. Two cases were excluded because only the Hematoxylin and Eosin (H&E) stained slide was provided, resulting in 68 patients with complete data. Among these 68 patients, we could successfully conduct an automated Ki67 LI in 47 patients as we excluded 21 patients due to abundant background staining in Ki67 or poor slide quality, hindering automated Ki67 LI determination.

The groups of included (*n* = 47) and excluded (*n* = 23) cases showed no significant differences in age (*p* = 0.055), tumor size (*p* = 0.505), tumor stage (*p* = 0.950) and manual Ki67 LI (*p* = 0.505).

### 3.1. Patient and Tumor Characteristics

Patient age ranged from 19 to 74 years (median 51 years, *n* = 47). The majority of patients was female (37/47, 78.7%). According to the European Network for the Study of Adrenal Tumors (ENS@T) staging system, 14 out of 47 patients had a stage 1 tumor (29.8%), 25 had a stage 2 tumor (53.2%), and 8 had a stage 3 tumor (17.0%). The tumor diameter ranged from 1.5 to 23 cm (median 8, *n* = 47). The Weiss score was ≥3 in 46/47 cases. A total of 24 patients received mitotane treatment, while 23 were in the surveillance group. The characteristics of the 47 patients with successful Ki67 LI determination are shown in Table 1.

### 3.2. Ki67 Assessment

**Figure 1:**
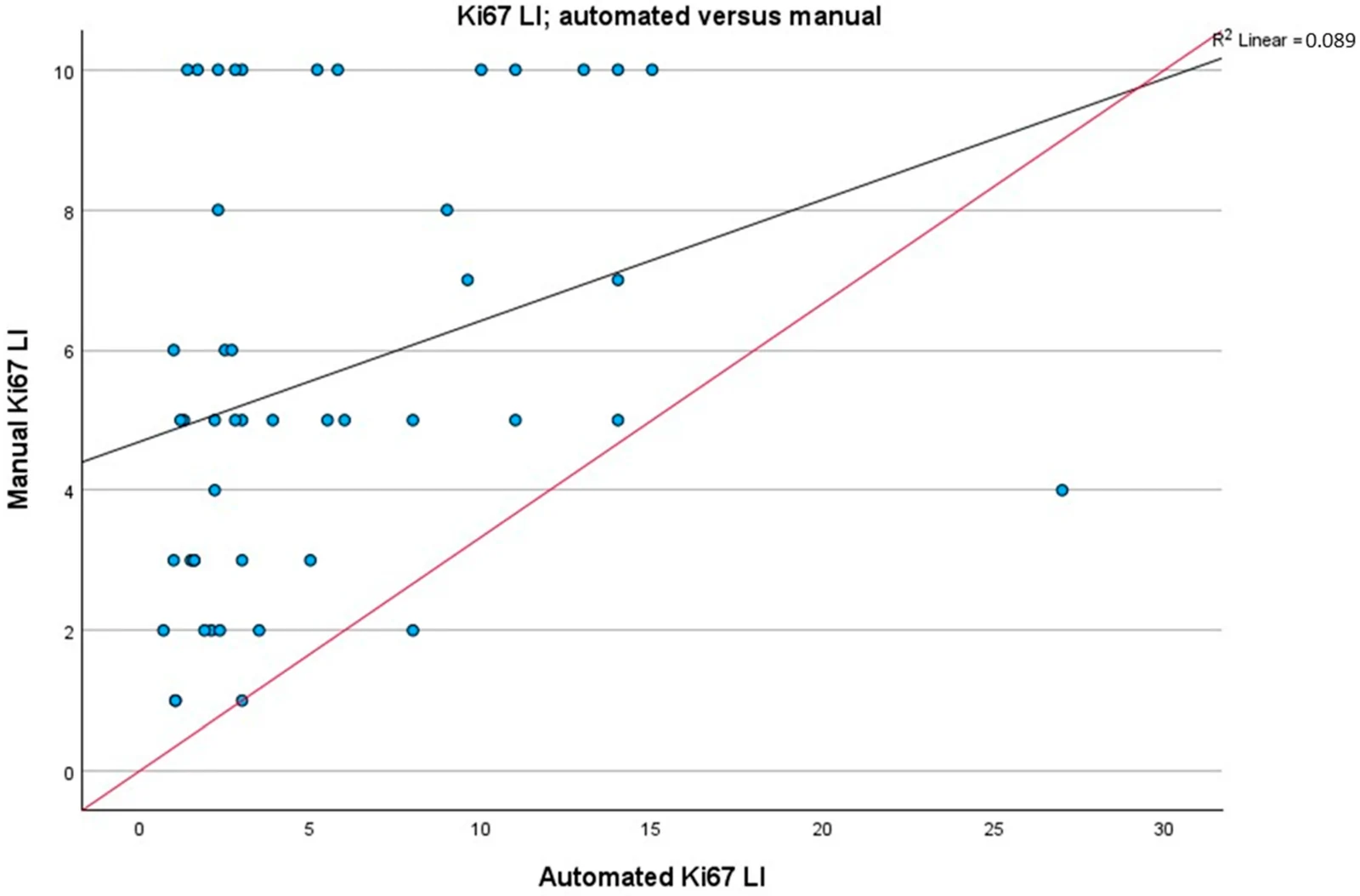
Scatterplot comparing the automated and manual Ki67 LI. The identity-line is depicted in red.

The average Ki67 LI based upon the assessment of local pathologists was 5.6% (*n* = 47, range 1–10), whereas the average Ki67 LI upon central review was 5.4% (*n* = 47, range 0.7–27; 95% CI: −1.30–1.82, not significant). Figure 1 illustrates the correlation between the automated and manual Ki67 LI. Interestingly, among the 47 patients analyzed, 8 (17%) had an automated Ki67 LI > 10% (the original study’s inclusion cut-off). Within this subgroup, the median age was 55 years, 25% were male, the median tumor size was 16 cm, and stage II and III tumors were present in 75% and 25% of patients, respectively. Six of these eight patients received adjuvant mitotane. Notably, only one true outlier was identified: a stage III patient with an automated Ki67 LI of 27% who was allocated to adjuvant mitotane, experienced early tumor recurrence, and died 14 months post-diagnosis—a clinical course typical of highly aggressive ACC.

**Figure 2:**
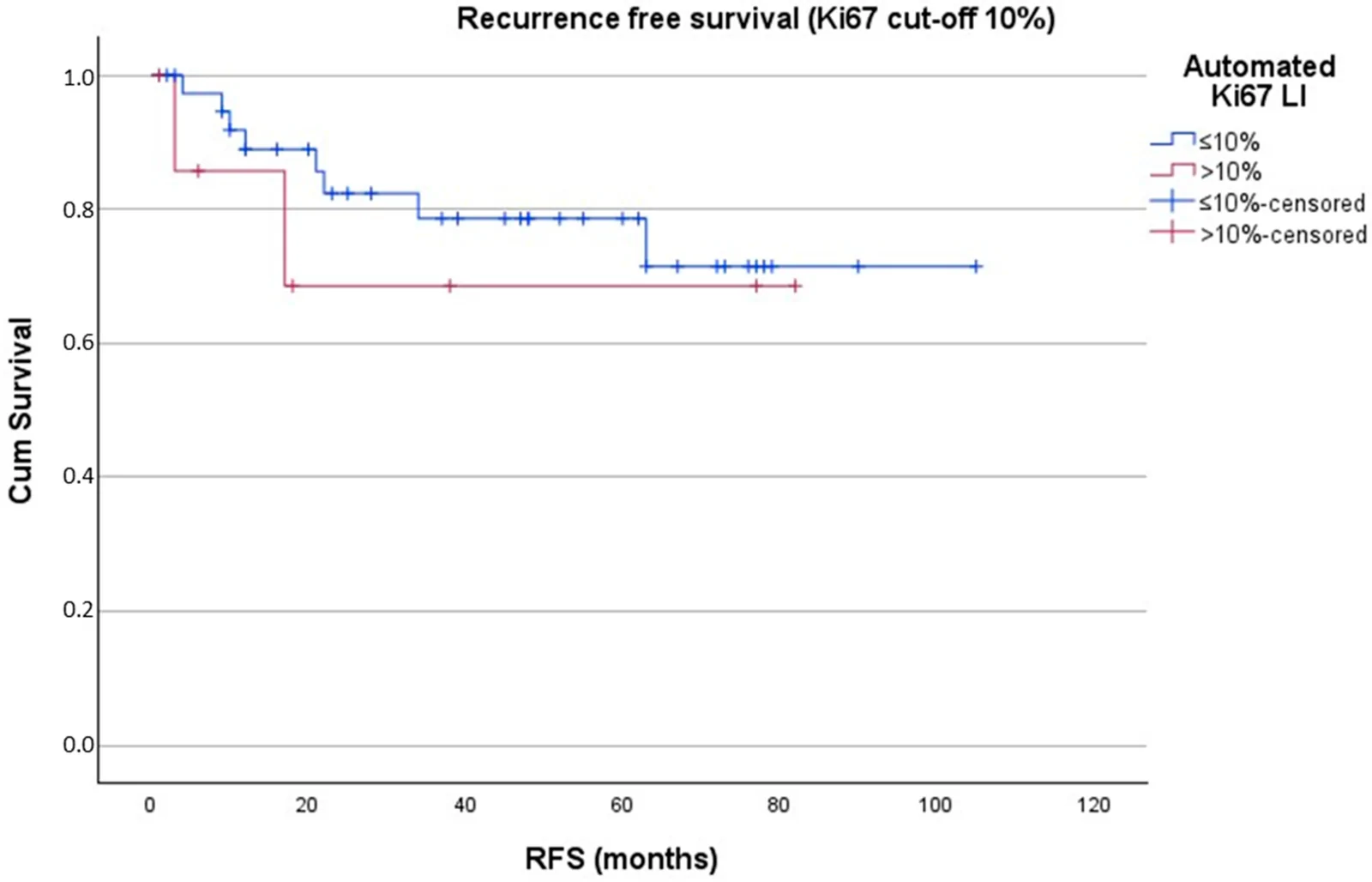
RFS in patients with an automated Ki67 LI above 10% (red line) and below 10% (blue line).

We subsequently compared the outcomes of the 47 patients with the provided data. Recurrence occurred in 10 patients (21.3%), of which three died of disease (6.4%). The median follow-up time for RFS was 37 months (*n* = 47, range 1–105). In the group with an automated Ki67 LI > 10%, the recurrence rate was 25.0% (2/8; including 2/5 patients on mitotane) and in the group with Ki67 LI ≤ 10% 20.5% (8/39; including 2/19 on mitotane). Accordingly, RFS was slightly lower, but not significantly different between patients with an automated Ki67 LI > 10% compared to those with Ki67 LI ≤ 10% (HR 1.66; 95% CI 0.35–7.86; *p* = 0.521 Figure 2; HR adjusted for adjuvant mitotane 1.77; 95% CI 0.37–8.63). Of note, if we analyze Ki67 LI as a continuous variable, there is a significant impact on RFS (adjusted HR 1.21; 95% CI 1.07–1.37; *p* = 0.003). However, in accordance with the main study result, the impact of adjuvant mitotane is not significant (HR adjusted for Ki67 LI: 0.67; 95% CI 0.13–3.33).

When comparing the death rate, 1 out of 8 patients died in the group with automated Ki67 LI > 10%, versus 2 out of 39 in the group with a Ki67 LI ≤ 10% (12.5% versus 5.1%, respectively, HR adjusted for adjuvant mitotane 2.80; 95% CI 0.24–32.61)).

In the group with an automated Ki67 LI ≤ 10% (*n* = 39), 36% was stage 1, 49% stage 2 and 15% stage 3. In the group with an automated Ki67 LI > 10% (*n* = 8), 75% was stage 2 and 25% stage 3. This difference was not significant (*p* = 0.106).

However, when analyzing tumor diameter, significantly larger tumors were observed in the group with an automated Ki67 LI > 10% (median 7.0 cm [IQR 7–10] vs. 16.0 cm [IQR 10.6–17], *p* = 0.003). Due to too few events in RFS and OS, multivariate analysis could not be performed.

## 4. Discussion

The aim of this study was to examine whether automated Ki67 LI reading shows better performance compared to manual reading in assessing the risk of recurrence in patients with ACC who underwent complete tumor resection. Specifically, we took advantage of the structure of the ADIUVO trial, the first randomized study evaluating adjuvant treatment with mitotane in patients with low-grade ACC, defined by a Ki67 LI of ≤10% [18]. One key finding of the ADIUVO trial was that such patients with low-grade ACC had a better prognosis than previously expected, in terms of RFS and OS, without any statistically significant difference between patients who received adjuvant mitotane treatment or active surveillance.

To strengthen this finding, the reliability of Ki67 LI as a prognostic marker and its role in risk stratification was further examined using an automated reading to overcome interobserver variability [20,21].

Ki67 is an immunohistochemical marker that stains the Ki67 protein, present in the nuclei of cycling cells, making it a widely used proliferation marker. In ACC, a Ki67 LI up to 10% is associated with better prognosis [1,22]. Nowadays, digital pathology systems facilitate automated Ki67 LI reading, which has been validated across various malignancies, reducing inter-observer discordance and standardizing Ki67 scoring protocols [23,24].

In the present study, the Ki67 LI obtained by automated image analysis was comparable to that derived from manual counting. Eight cases demonstrated an automated Ki67 LI greater than 10%; however, only one case showed a marked discrepancy between automated and manual assessment (27% versus 4%). This was a patient with an aggressive course of disease, more typical of an aggressive tumor. Therefore, this ACC was misdiagnosed as a low-grade tumor by the local pathologist using manual reading.

Automated Ki67 LI assessment was superior in prognostic stratification in only one case, and we observed an overall good concordance with the manual assessment. This is explainable considering that pathologists from expert centers have been involved in the ADIUVO trial. Discordances between automated versus manual Ki67 assessment can be explained by differences in pathologist selection of hotspot areas of Ki67 labeling and the counting itself. In only a few patients, the automated Ki67 LI was slightly above 10%, with readings ranging from 11% to 15%. However, we did not find any difference in survival between patients with automated Ki67 LI ≤ 10% or above this cut-off, and survival of patients who received mitotane or surveillance were not significantly different. These findings are reassuring in terms of lack of influence on the outcomes of the ADIUVO trial [17]. In addition, our data suggest a potential correlation between Ki67 LI and tumor size.

Interestingly, a recent retrospective study from a Spanish registry, including a total of 244 patients, showed a benefit of adjuvant mitotane treatment on RFS in a pooled analysis (including tumors with all Ki67 scores), but no benefit in a subgroup analysis, including tumors with a Ki67 LI of <10% [25].

The main limitation of our study derives from the impossibility of retrieving adequate material from all the patients included in the ADIUVO trial and this resulted in small numbers in some subgroups that hindered statistical analysis. Furthermore, 21 cases exhibited extensive background staining, which precluded reliable automated Ki67 LI quantification and necessitated their exclusion from the study. However, this fact also points to a limitation of automated Ki67 LI reading. While the exact etiology of this artifact remains uncertain, it may be attributed to older unstained slides, suboptimal formalin fixation or other pre-analytical processing issues. Although our staining protocols are optimized for internal processing standards, variations in tissue handling can nonetheless impact results.

For future multicenter studies, centralizing automated Ki67 counting or other relevant algorithms should be considered to ensure methodological consistency, unless identical algorithms are available across the participating centers. Furthermore, emerging AI-based technologies and algorithms may also improve the accuracy of Ki67 assessment through automated hotspot selection, thereby eliminating potential pathologist-related bias in hotspot selection [26].

## 5. Conclusions

In conclusion, our findings confirm Ki67 LI as a robust predictor of recurrence post-ACC extirpation. While manual assessment by expert pathologists remains highly reliable and avoids the logistical delays associated with automated scoring, centralized digital analysis offers enhanced standardization and may identify clinically relevant outliers. Consequently, manual Ki67 LI remains a valid and practical method for future clinical trials.

## Figures and Tables

**Table 1:** Baseline characteristics of the 47 included patients.

Characteristics	
**Age at diagnosis (years)**	
Median, range	51 (19–74)
**Sex** n, (%)	
Male	10 (21.3%)
Female	37 (78.7%)
**Stage** N, (%)	
Stage 1	14 (29.8%)
Stage 2	25 (53.2%)
Stage 3	8 (17.0%)
**Tumor diameter (cm)**	
Median, range	8 (1.5–23)
**WEISS score** n, (%)	
<3	1 (2.1%)
≥3	46 (97.9%)
**Ki67 LI**	
Mean ± SD, manual	5.6 ± 3.1%
Mean ± SD, automated	5.4 ± 5.3%
Median, manual	5 [IQR 3–9]
Median, automated	3 [IQR 1,8–8]
**Treatment** n, (%)	
Mitotane	24 (51%)
Surveillance	23 (49%)

## Data Availability

All data generated or analyzed during this study are included in this published article.
